# Torque Teno Virus Load Predicts Opportunistic Infections after Kidney Transplantation but Is Not Associated with Maintenance Immunosuppression Exposure

**DOI:** 10.3390/biomedicines11051410

**Published:** 2023-05-09

**Authors:** Lucía Cañamero, Adalberto Benito-Hernández, Elena González, Clara Escagedo, María Rodríguez-Vidriales, María del Mar García-Saiz, Rosalía Valero, Lara Belmar, María Angeles de Cos, María Victoria Francia, Juan Carlos Ruiz, Emilio Rodrigo

**Affiliations:** 1 Immunopathology Group, Nephrology Department, Marqués de Valdecilla University Hospital-IDIVAL, University of Cantabria, 39012 Santander, Spain; 2 Immunopathology Group, Immunology Department, Marqués de Valdecilla University Hospital-IDIVAL, University of Cantabria, 39012 Santander, Spain; 3 Clinical Pharmacology Department, Marqués de Valdecilla University Hospital-IDIVAL, University of Cantabria, 39012 Santander, Spain; 4 Infectious Diseases and Clinical Microbiology Group, Marqués de Valdecilla University Hospital-IDIVAL, University of Cantabria, 39011 Santander, Spain; mvictoriafrancia@scsalud.es

**Keywords:** acute rejection, immunosuppression, infection, kidney transplantation, monitoring, Torque Teno virus

## Abstract

Measuring the non-pathogenic Torque Teno Virus (TTV) load allows assessing the net immunosuppressive state after kidney transplantation (KTx). Currently, it is not known how exposure to maintenance immunosuppression affects TTV load. We hypothesized that TTV load is associated with the exposure to mycophenolic acid (MPA) and tacrolimus. We performed a prospective study including 54 consecutive KTx. Blood TTV load was measured by an in-house PCR at months 1 and 3. Together with doses and trough blood levels of tacrolimus and MPA, we calculated the coefficient of variability (CV), time in therapeutic range (TTR) and concentration/dose ratio (C/D) of tacrolimus, and the MPA-area under the curve (AUC-MPA) at the third month. TTV load at the first and third month discriminated those patients at risk of developing opportunistic infections between months 1 and 3 (AUC-ROC 0.723, 95%CI 0.559–0.905, *p* = 0.023) and between months 3 and 6 (AUC-ROC 0.778, 95%CI 0.599–0.957, *p* = 0.028), respectively, but not those at risk of acute rejection. TTV load did not relate to mean tacrolimus blood level, CV, TTR, C/D and AUC-MPA. To conclude, although TTV is a useful marker of net immunosuppressive status after KTx, it is not related to exposure to maintenance immunosuppression.

## 1. Introduction

Kidney transplantation is the best therapy for patients with end-stage renal disease. While in liver transplantation a significant number of patients can be withdrawn immunosuppressive drugs due to the development of variable grades of tolerance, most of the other solid organ transplants need to maintain the immunosuppressive drugs while the graft is functioning to avoid alloimmune response [1,2]. Routine follow-up of kidney grafts for transplant physicians implies maintaining appropriate combinations of drugs and drug doses that are known that reduce the appearance of rejection. On the other hand, this immunosuppressive therapy is also associated with undesirable side effects such as infections, cancer, metabolic diseases, and cardiovascular events that increase mortality risk after solid organ transplantation [3,4,5]. Traditionally, it has been thought that transplant physicians should establish an adequate balance between over- and under-immunosuppression to avoid, at the same time, these side effects and the alloimmune response. Unfortunately, infection and rejection can appear at the same time [6]. Common practice is to monitor calcineurin-inhibitor (CNI) trough blood levels, trying to keep them inside a narrow therapeutic range, although different studies have highlighted that these levels correlate more closely with the risk of side effects than with the rejection risk [7,8].

To improve the results of kidney transplantation, it is important to have new, minimally invasive monitoring methods that would make it possible to assess the risk of both infection and rejection. Some of these methods, such as the ability to activate T helper lymphocytes, have already been used in clinical trials for monitoring liver transplantation, although their value has not been sufficiently proven [9]. Recently, a new promising method that allows estimating the net immunosuppressive state by measuring the Torque Teno Virus (TTV) load has been developed. TTV is a member of the Anelloviridae family that are ubiquitous and non-pathogenic for humans [10]. Previous studies in kidney transplant recipients have shown that a high TTV load relates to a higher risk of infection and other immunosuppressive-related side effects [11,12,13,14,15,16,17,18], whereas low TTV loads are associated with a higher risk of acute rejection [14,16,17,18,19,20,21,22,23]. In this sense, by measuring TTV in blood, we can assess if a kidney transplant recipient is over- or under-immunosuppressed and guide the immunosuppressive therapy.

So far, it is not known precisely which immunosuppressive-related variables influence TTV levels. Some studies have linked higher doses of antimetabolite or CNI drugs or higher CNI blood levels to higher blood TTV loads, but this has not been demonstrated in all studies [13,19,24,25,26]. Van Rijn et al. reported that patients treated with tacrolimus showed higher TTV loads than those under cyclosporine [21]. On the other hand, it has not been previously analyzed whether mycophenolic acid exposure measured by calculating the area under the curve (AUC-MPA) or whether continuous tacrolimus exposure influences TTV burden. The area under the curve is the gold standard to know the appropriate exposure to mycophenolic and relates better to rejection risk and side effect development than mycophenolic trough levels [27]. Related to CNI, some studies support that continuous exposure measured as coefficient of variability (CV), time in therapeutic range (TTR), or concentration/dose ratio (C/D) relates better than the simple trough CNI blood level measurement to rejection and side effect risk [28,29,30,31]. We hypothesized that TTV load is associated with exposure to mycophenolate mofetil and CNI.

## 2. Materials and Methods

This prospective study was conducted following the guidelines of the Declaration of Helsinki and approved by the Regional Ethics Committee in our institution (reference number: PI20/01710; 22 December 2020). A total of 54 consecutive kidney transplants from deceased donors performed in our center from January 2021 to April 2022 were recruited after giving written consent prior to kidney transplant. Recipients from non-controlled cardiac death donation, with preformed donor specific antibodies and highly hypersensitized with a panel reactive antibody over 98% were excluded.

Relevant information about recipient, donor, and transplant characteristics was collected. All acute rejection episodes were biopsy proven. Indication biopsies were performed when the level of creatinine increased by 25% or more over its previous value or when proteinuria persisted >1 g per day. We considered opportunistic infections those related to cellular and humoral immunosuppression, which included viral (CMV, EBV, HSV, VZV, BK polyomavirus associated nephropathy and relapsed hepatitis HBV, HCV), bacterial (typical and atypical mycobacterium, others as Nocardia, Listeria, etc.), and fungal (*Pneumocystis jirovecii* pneumonia, Candida and invasive fungal infections such as Aspergillus) infections. Standard microbiology techniques were used for the detection of viral, bacterial and fungal infections.

Maintenance immunosuppressive therapy consisted of twice daily tacrolimus, mycophenolate mofetil, and prednisone. Recipients of organs from expanded criteria donors and at risk of delayed graft function received induction therapy with basiliximab. Thymoglobulin was used as induction therapy when patients had a higher risk of rejection due to hypersensitization or previous graft loss due to acute rejection. All patients received prophylaxis with trimethoprim-sulfamethoxazole for 6 months after transplantation and with valganciclovir for 3 months in CMV IgG-negative recipients of a CMV IgG-positive organ and in patients receiving thymoglobulin induction.

Whole-blood concentrations (µg/L) of tacrolimus were determined by chemiluminescent microparticle immunoassay on the Architect iSystem (CMIA; Abbott Laboratories, Abbott Park, IL, USA). This immunoassay is designed to have a precision of ≤10% total coefficient of variation (CV), a mean recovery of 100 ± 10% of the expected value, a limit of detection of ≤1.5 µg/L, and a functional sensitivity of ≤2 µg/L. All levels of tacrolimus up to day 90 were collected. The tacrolimus target trough blood levels up to month 3 were 8 to 12 ng/mL. The variability of tacrolimus blood levels was estimated by means of the coefficient of variation (CV) calculated according to the following equation:

CV (%) = (σ/μ) × 100(1)

where σ is the standard deviation, and μ is the mean tacrolimus concentration of all available samples [28]. The percent of the time in the therapeutic range (8 to 12 ng/mL) and above 12 ng/mL were calculated using the Rosendaal method [30]. Tacrolimus C/D ratios were calculated at months 1 and 3. Fast metabolizers were defined by a tacrolimus level-dose ratio <1.05 [31].

Trough blood concentrations of mycophenolic acid (MPA) in human plasma (mg/L), were quantified by homogenous enzyme immunoassay (Emit 2000 Mycophenolic Acid Assay; Siemens, Munich, Germany) at months 1 and 3. This immunoassay is designed to have a precision of ≤10% total CV, and a limit of detection of ≤0.1 mg/L. The dynamic range of this immunoassay is from 0.1 mg/L to 15 mg/L based on the analytical sensitivity of the assay. If the analysis of a patient sample is outside the calibration range, it is diluted, allowing it to determine the concentration. No cross-reactivity with MPAG (mycophenolic acid glucuronide), the main metabolite of MPA, or with cyclosporine or tacrolimus has been observed.

The full mycophenolic acid area under the curve (AUC-MPA) was calculated at month 3 using an abbreviated procedure of drawing blood samples at time 0, at 30 min and 2 h after having taken the drug, according to the procedure previously reported by Pawinski et al. The regression equation for AUC_0–12h_ estimation that gave the best performance for this model was: 7.75 + 6.49.C_0h_ + 0.76.C_0.5h_ + 2.43.C_2h_ [32].

Samples for measuring TTV load were drawn before transplantation and at days 30 and 90 post-transplantation. Briefly, the blood obtained by venipuncture with a BD Vacutainer K2E 5.4 mg tube, cat #368856 (BD Vacutainer, Franklin Lakes, NJ, USA) was centrifuged at 1.300 rpm and the plasma was frozen at −80 °C until processing. Free viral DNA was purified from 400 µL of plasma from all specimens using the QIAamp MinElute Virus Spin Kit Cat. # 57704 (Qiagen GmbH, Hilden, Germany) as specified by the manufacturer. The presence and viral load of TTV in the samples were determined in duplicate using a previously described TaqMan (TM)-PCR assay human TTV APP2XDMP (ThermoFisher, Life technologies, Paisley, UK) in a StepOnePlus Real-Time PCR System (AB Applied Biosystems, Singapore). This assay is based on the specific amplification of a highly conserved viral segment in the untranslated region of TTV, which has the potential for sensitive and specific detection of all TTV genotypes present in GenBank [33]. The procedures used for copy number quantification and assessment of specificity, sensitivity, intra-, and inter-assay precision, and reproducibility have been previously described [34,35]. The lower limit of sensitivity was 1.0 × 10^3^ viral genomes per ml of plasma sample. This protocol has recently been compared and validated against the commercial TTV R-GENE^®^ kit (bioMérieux, Craponne, France) [18].

As a positive control, a 143 bp PCR fragment from the same untranslated region of TTV genome (NC_015783.1) was amplified using the primers TTV Sen (5′ GTGCCGTAGGTGAGTTTA 3′) and TTV AntisL (5′ ATGGACCGGCGGTCTCCACGG 3′) and cloned into the pCR™2.1 cloning vector (TA Cloning™ Kit, # K202040 ThermoFisher, Invitrogen Carlsbad, CA 92008 USA). The resulting plasmid was then purified with QIAprep Spin Miniprep Kit, # 2710 (Qiagen GmbH, Hilden, Germany) and quantified using Nanodrop 2000C spectrophotometer ThermoFisher Scientific # ND-2000C (Thermo Fisher Scientific, DE 19810 USA). The standard curve was established with the points A = 1.0 × 10^12^ copies, B = 1.0 × 10^10^ copies, C = 1.0 × 10^8^ copies, D = 1.0 × 10^6^ copies, E = 1.0 × 10^4^ copies, F = 1.0 × 10^2^ copies, G = 1.0 copies, H = 0 copies.

Continuous variables were expressed as mean ± standard deviation if normally distributed or as median and interquartile range (IQR) if non-normally distributed. Categorical variables were described as relative frequencies. The Spearman’s rank correlation coefficient was used to explore the relationship between TTV load and continuous variables. The Wilcoxon rank test was used for comparing TTV loads at different time points. The Mann–Whitney U test was used to compare TTV load differences among dichotomous variables. The ability of TTV load to discriminate infection and rejection was analyzed by constructing receiver operating characteristic (ROC) curves. Univariate and multivariate logistic regression analyses were used to analyze the relationship between TTV load and infection and rejection. A *p*-value less than 0.05 was considered statistically significant. Statistical analyses were performed with SPSS, version 15.0 (SPSS, Inc, Chicago, IL, USA).

## 3. Results

The main patient characteristics are shown in Table 1. Fifty-four kidney transplant patients were included and followed throughout the first year. A total of 1 patient died due to respiratory sepsis at month 10. Biopsy-proven acute rejection was diagnosed in eight patients during the first post-transplant month, in four patients between first and third post-transplant months and in only one between months 3 and 6. A total of 10 (18.5%; 9 CMV infections and 1 biopsy-proven BK polyomavirus nephropathy) and 6 (11.1%; 5 CMV infections and 1 biopsy-proven BK polyomavirus nephropathy) patients experienced at least one opportunistic infection between months 1 and 3 and between months 3 and 6, respectively.

**Figure 1:**
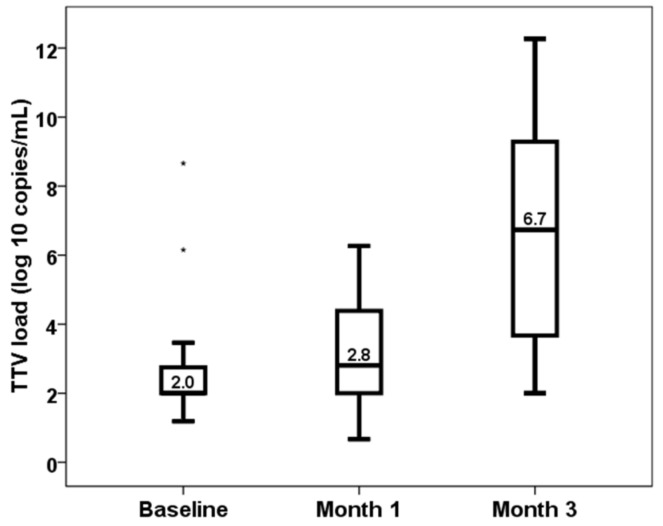
Kinetics of TTV load (log_10_ copies/mL).

Median TTV load values increased from pretransplant (2.00, IQR 0.76) to month 1 (2.81, IQR 2.41, *p* < 0.001) and month 3 (6.73, IQR 5.74, *p* < 0.001) (Figure 1). Baseline TTV load did not relate to recipient age (rho = 0.177, *p* = 0.200), to donor age (rho = 0.108, *p* = 0.435) and to time in renal replacement therapy (rho = 0.091, *p* = 0.513) according to Spearman correlation analysis. Baseline TTV load was significantly higher in male recipients (median 2.00, IQR 0 vs. median 2.47, IQR 0.98, Mann–Whitney *p* = 0.019). Neither CMV serostatus (median 2.00, IQR 0.87 vs. median 2.00, IQR 0.75, Mann–Whitney *p* = 0.453) nor preemptive transplant (median 2.19, IQR 0.75 vs. median 2.00, IQR 0.77, Mann–Whitney *p* = 0.171) showed significantly different baseline TTV loads.

Spearman correlations between TTV load at months 1 and 3 and continuous variables are shown in Table 2 and Table 3, respectively. In patients who received thymoglobulin induction, TTV loads at month 1 (rho −0.041, *p* = 0.890) and at month 3 (rho −0.326, *p* = 0.256) were not associated with the accumulated thymoglobulin dose. The Mann–Whitney U test did not find significant differences in TTV load at month 1 comparing recipient gender (male) (2.10, IQR 2.66 vs. 2.92, IQR 2.30, *p* = 0.872), diabetic nephropathy (2.94, IQ 2.54 vs. 2.23, IQR 2.25, *p* = 0.401), retransplant (2.81, IQR 2.41 vs. 2.87, IQR 3.09, *p* = 0.896), preemptive transplantation (2.91, IQR 2.54 vs. 2.00, IQR 2.39, *p* = 0.362), induction use (2.57, IQR 2.66 vs. 2.96, IQR 2.37, *p* = 0.936), thymoglobulin (2.57, IQR 2.30 vs. 3.35, IQR 2.64, *p* = 0.258), any tacrolimus level <5 at month 1 (2.66, IQR 2.45 vs. 3.15, IQR 2.45, *p* = 0.883), any tacrolimus level <6 at month 1 (2.57, IQR 2.33 vs. 3.79, IQR 3.40, *p* = 0.205) and fast tacrolimus metabolizers (4.39, IQR 2.56 vs. 2.57, IQR 2.17, *p* = 0.197).

By the Mann–Whitney U test, we did not find significant differences in TTV load at month 3 comparing recipient gender (5.98, IQR 6.12 vs. 6.73, IQR 5.31, *p* = 0.333), diabetic nephropathy (6.95, IQR 6.74 vs. 6.34, IQR 4.11, *p* = 0.839), retransplant (6.74, IQR 5.73 vs. 5.98, IQR 7.80, *p* = 0.802), preemptive transplantation (6.78, IQR 6.00 vs. 5.43, IQR 4.64, *p* = 0.420), thymoglobulin (6.63, IQR 5.61 vs. 7.77, IQR 5.94, *p* = 0.286), any tacrolimus level < 5 at month 3 (6.51, IQR 5.95 vs. 6.87, IQR 5.60, *p* = 0.157), any tacrolimus level < 6 at month 3 (7.49, IQR 5.89 vs. 6.59, IQR 6.87, *p* = 0.961) and fast tacrolimus metabolizers (7.78, IQR 3.84 vs. 6.63, IQR 6.20, *p* = 0.381). Induction use (3.47, IQR 4.10 vs. 7.53, IQR 4.98, *p* = 0.008) was the only immunosuppression-related variable associated with the TTV load at month 3.

**Figure 2:**
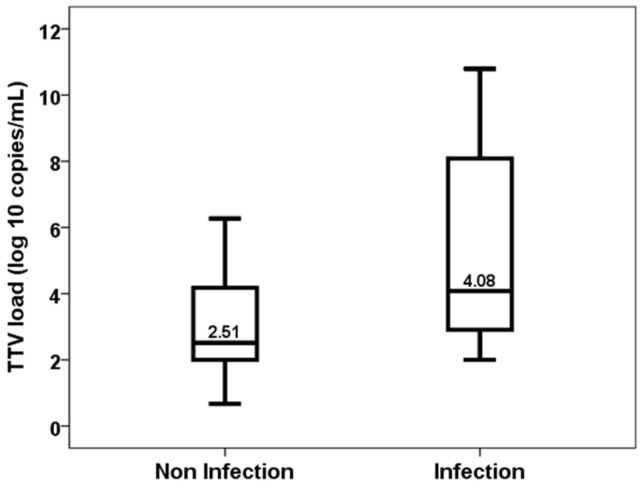
TTV load at first month in patients with and without an opportunistic infection from month 1 to 3.

**Figure 3:**
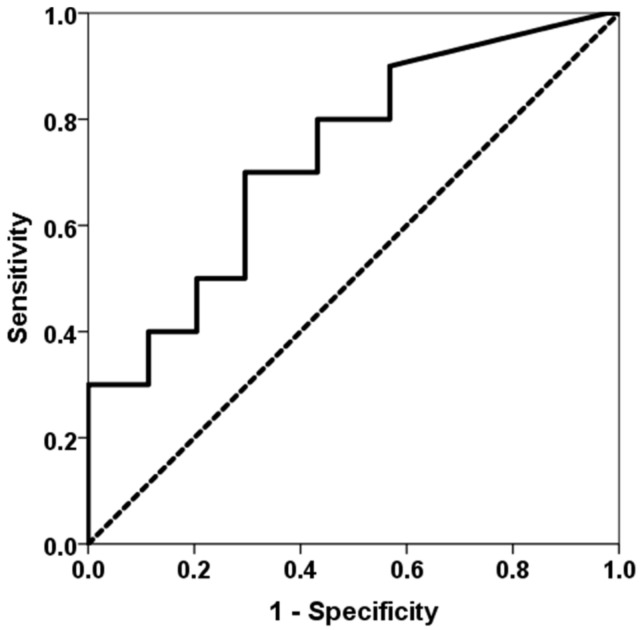
ROC curve of TTV load at first month for predicting an opportunistic infection between months 1 and 3. The solid line represents the ROC curve (AUC = 0.723). The dotted line represents the reference ROC curve with AUC of 0.50.

Patients who developed an opportunistic infection between months 1 and 3 showed higher levels of TTV load at first month (2.51, IQR 2.22 vs. 4.08, IQR 5.57, *p* = 0.020) (Figure 2). TTV load at first month was able to discriminate those patients at risk of developing opportunistic infections between months 1 and 3 (AUC-ROC 0.723, 95%CI 0.559–0.905, *p* = 0.023) (Figure 3). By univariate logistic regression, TTV load at month 1 related to a higher risk of an opportunistic infection from months 1 to 3 (OR 1.682, 95%CI 1.134–2.495, *p* = 0.010). After multivariate logistic regression analysis adjusting for other variables related to TTV load (recipient age, donor age, estimated GFR, diabetes mellitus and recipient female), TTV load at month 1 remained independently associated with a higher risk of an opportunistic infection from months 1 to 3 (OR 1.682, 95%CI 1.134–2.495, *p* = 0.010).

**Figure 4:**
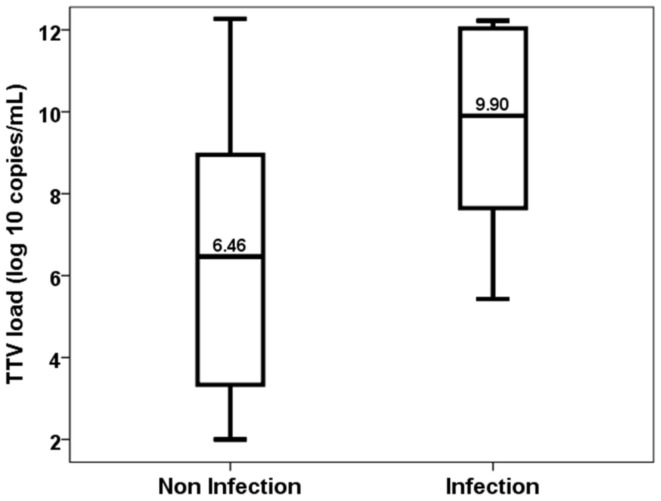
TTV load at the third month in patients with and without an opportunistic infection from month 3 to 6.

**Figure 5:**
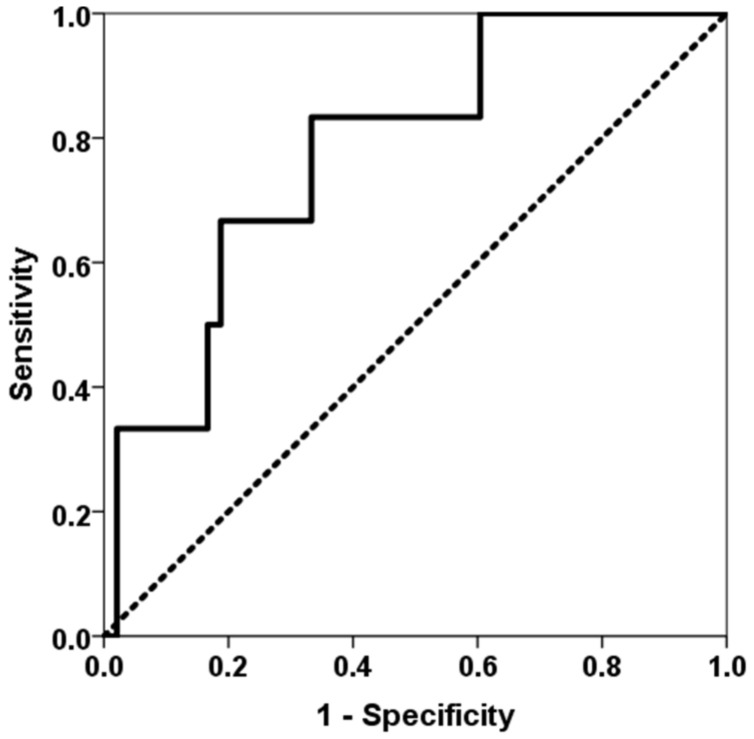
ROC curve of TTV load at the third month for predicting an opportunistic infection between months 3 and 6. The solid line represents the ROC curve (AUC = 0.778). The dotted line represents the reference ROC curve with AUC of 0.50.

Similarly, patients who developed an opportunistic infection between months 3 and 6 showed higher levels of TTV load at the third month (6.46, IQR 5.74 vs. 9.90, IQR 4.99, *p* = 0.026) (Figure 4). TTV load at the third month discriminated those patients at risk of developing opportunistic infections from months 3 to 6 (AUC-ROC 0.778, 95%CI 0.599–0.957, *p* = 0.028) (Figure 5). TTV load at month 3 related to a higher risk of an opportunistic infection from months 3 to 6 (OR 1.444, 95%CI 1.017–2.050, *p* = 0.040) and this relationship remained significant (OR 1.444, 95%CI 1.017–2.050, *p* = 0.040) after adjusting by confounders variables such as recipient age, donor age, female recipient gender, estimated GFR and diabetes mellitus.

**Figure 6:**
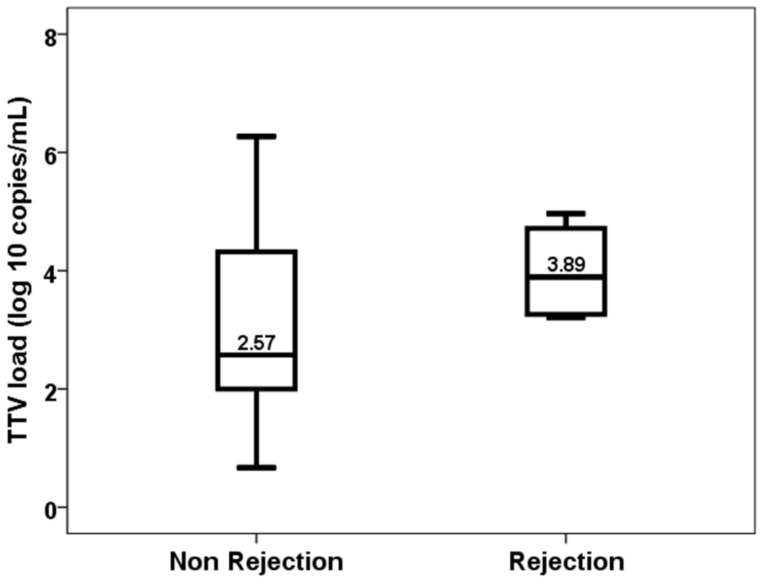
TTV load at the first month in patients with and without acute rejection from month 1 to 3.

By contrast, TTV load at the first month was not significantly higher in patients who developed acute rejection between months 1 and 3 (2.57, IQR 2.33 vs. 3.89, IQR 1.61, *p* = 0.177) (Figure 6) and was not useful to discriminate these patients (AUC-ROC 0.710, 95%CI 0.565–0.855, *p* = 0.165).

## 4. Discussion

After transplantation, TTV load increased in month 1 and to higher levels in month 3. Similar to us, most authors analyzing kinetic changes in TTV load have reported that TTV load increases after transplantation reaching its maximal value at months 3 to 6 [13,14,17,36,37]. In this sense, an optimal point to measure TTV load would be at month 3 post-transplantation, although we detected that both TTV loads at months 1 and 3 related to a higher risk of an opportunistic infection. This relationship between TTV load and later infection has been clearly demonstrated by most but not all authors [11,12,13,14,15,16,17,18,21,37]. The AUC-ROC of TTV for predicting infection ranged between 0.580 and 0.650 [11,12,13,14,16,17,18]. We also reported that for each log 10 increase in TTV load at month 1 the risk of an opportunistic infection increased by 68% and at month 3 increased by 44%. This result is concordant with other studies in which the increase in infection risk ranged from 6% to 188% per 1 log of TTV load, depending on the infection definition [13,14,17,18]. Pooled data from 16 studies have shown that the risk of infection increased by 16% per 1 log of TTV load increase [23]. Being an opportunistic infection more specifically related to over-immunosuppression, it is expected that the relationship between TTV load and an opportunistic infection will be stronger than with a global infection, as previously reported by Doberer et al. [17]. Of note, TTV load is associated with higher infection-related death even beyond the first-year post-transplantation [38].

Due to the low number of rejection episodes in our study, we did not find a relationship with TTV load, although the median differences between patients with (3.89 log) and without rejection (2.57 log) were striking. Most previous studies have found that patients with lower TTV loads are at a higher risk of acute rejection [14,16,17,18,19,20,21,23], antibody-mediated rejection [19] and subclinical rejection [22]. TTV load has a discriminative ability for predicting acute rejection with AUC-ROC ranging from 0.73 to 0.82 [17,18,20]. Collecting data from 15 studies, the meta-analysis by Van Rijn et al. concluded that the risk of rejection decreased by 10% per 1 log of TTV load [23]. The fact that rejection can be diagnosed in a more homogeneous way than infection thanks to the Banff consensus among the different centers could facilitate more concordant results in the different studies.

The only immunosuppressive-drug variable related to TTV load was induction use. Those patients who have received induction showed higher TTV loads. Although the use of thymoglobulin did not relate statistically to TTV load, there was a trend in the median TTV load between those treated and non-treated with lymphocyte-depleting antibodies as induction. Induction with antibodies and/or thymoglobulin is the immunosuppressive treatment most consistently related to higher TTV loads, since it is associated with a stronger immunosuppressive effect, although this relationship has not been confirmed by all authors [13,14,19,36].

The main finding of our study was that higher exposure to maintenance immunosuppressive drugs such as tacrolimus, mycophenolate mofetil, or prednisone was not associated with a higher TTV load. We conducted a detailed study of global tacrolimus exposure. To this end, we collected variables related to sustained exposure to tacrolimus that had not been previously studied in relation to TTV burden. The tacrolimus CV has been related to a worse outcome in solid organ transplantation, not only in relation to under-immunosuppression and increased risk of rejection, but also in relation to over-immunosuppression and increased risk of infections [28,29]. A shorter TTR has also been related to a worse evolution of renal transplantation, with more rejection, renal graft loss, and infections [30]. A lower tacrolimus level-value dose ratio is related to rapid metabolizers who have more difficulty in maintaining stable levels, which puts them at risk of changes in the level of immunosuppression [31]. We found no relationship between any of these tacrolimus exposure variables and TTV burden. We also did not find that the mean tacrolimus levels were related to TTV load as in other studies in kidney transplantation [13,19,25], unlike Gorzer et al. in lung transplants [24].

Moreover, different from previous studies, we analyzed the relationship between TTV load and exposure to mycophenolic acid by measuring the gold standard AUC-MPA. Previous authors reported that MMF dose related to TTV load, but neither of them analyzed AUC-MPA [13]. There is strong evidence in favor of using AUC-MPA, rather than trough levels or no monitoring, to accurately assess the level of mycophenolic acid exposure to monitor kidney transplant recipients [27]. Difficulties in measuring AUC-MPA, which requires multiple blood samples over several hours, have made it not the standard practice in most centers. Unexpectedly, we found no relationship between mycophenolic exposure and TTV levels. In the same way, the prednisone dose was not related to TTV. This lack of relationship leads us to suspect that the overall levels of immunosuppression in patients depend to a large extent on factors not exclusively related to immunosuppressive treatment, such as nutritional status or frailty. The relationship of TTV with the infection, and, in previous studies, with the rejection, confirms that it is a biomarker of the state of immunosuppression and that it is not fully dependent on the maintenance immunosuppression.

In our study, male recipients showed higher baseline TTV loads. Previous studies reported a similar finding [13,19,36]. By contrast, we did not find any relationship between pretransplant TTV load and recipient age and CMV serostatus, having been these relationships previously reported [13,14,19,36]. Some variables that could be related to a certain degree of immunosuppression such as the time in renal replacement therapy and if the recipient received a preemptive transplantation were not related to pretransplant TTV load in our study [21]. At month 3 post-transplantation, we found that a higher donor age was associated with a higher TTV load. The relationship between donor age and TTV load has been reported by Strassl et al. [13]. Since TTV viral load increases with age, grafts from older donors transmit a higher TTV load with the graft itself, which could increase the long-term recipient blood TTV load.

The main limitation of our single-center study was the sample size. We demonstrated a relationship between the burden of TTV and opportunistic infections, but not with rejection, mainly due to its low incidence. Another limitation was that we used a non-standardized in-house PCR technique to measure the TTV burden. A recent publication has highlighted the agreement between in-house and standardized techniques and that both methods are useful for measuring TTV [39]. As an advantage, we performed a detailed prospective study on the global exposure of each patient to global immunosuppression. Being a prospective study allowed us to collect the incidence of infection and rejection in defined periods (1 to 3 months, 3 to 6 months) to determine the predictive capacity of each TTV value. Until now, each study has defined infection in a variable way and the times in which the risk of infection and rejection were analyzed in relation to the TTV have not been homogeneous. There is an ongoing randomized controlled trial comparing standard versus TTV-guided immunosuppression that will allow us to know in more detail the relationship between TTV and kidney transplant complications more accurately [40].

## 5. Conclusions

To conclude, we carried out a prospective study in which it was shown that the TTV loads at one month and the third month after transplantation were independently related to the subsequent risk of infection in renal transplantation. A trend was also detected where patients who experienced an acute rejection had lower levels of TTV. TTV viral load was related to induction use but was not related to overall exposure to maintenance immunosuppression. It is necessary to analyze in greater depth which variables determine the blood values of TTV to use it as a non-invasive biomarker useful to assess the global level of immunosuppression in kidney transplants and other solid organ transplants.

## Figures and Tables

**Table 1:** Main patient characteristics.

Number of patients	54
Recipient age (years)	57.8 ± 12.0
Recipient gender (% male)	74.1
Diabetic nephropathy (%)	27.8
Time in renal replacement therapy (months)	49 ± 100
Retransplant (%)	14.9
Preemptive transplantation (%)	20.4
Donor age (years)	54.0 ± 15.7
HLA mismatches	4.5 ± 1.1
Cold ischemia time (hours)	19.5 ± 6.6
Induction (%)	74.1
Thymoglobulin (%)	25.9
Delayed graft function (%)	27.8
First year acute rejection (%)	22.2
First month eGFR (mL/min/1.73 m^2^)	51 ± 21
First month albuminuria (mg/g)	159 ± 321
Third month eGFR (mL/min/1.73 m^2^)	53 ± 21
Third month albuminuria (mg/g)	105 ± 196
Month 1 prednisone dose (mg)	14.1 ± 2.4
TTR 8–12 at month 1 (%)	45 ± 24
TTR > 12 at month 1 (%)	49 ± 27
Mean tacrolimus level at month 1 (ng/mL)	12.7 ± 2.3
Any tacrolimus level < 5 at month 1 (%)	11.1
Any tacrolimus level < 6 at month 1 (%)	14.8
Coefficient of variability at month 1 (%)	29.1 ± 12.2
Tacrolimus trough level/Dose at month 1	1.8 ± 0.9
Month 3 prednisone dose (mg)	7.4 ± 1.6
TTR 8–12 at month 3 (%)	59 ± 21
TTR > 12 at month 3 (%)	30 ± 18
Mean tacrolimus level at month 3 (ng/mL)	11.6 ± 1.4
Any tacrolimus level < 5 at month 3 (%)	14.8
Any tacrolimus level < 6 at month 3 (%)	27.8
CV at month 3 (%)	31.1 ± 10.0
C/D at month 3	2.3 ± 1.3
Fast tacrolimus metabolizers (%)	14.8
AUC-MPA at month 3 (µg × h/mL)	40.2 ± 14.6

eGFR: estimated glomerular filtration rate; TTR: time in therapeutic range; CV: coefficient of variability of tacrolimus; C/D: concentration/dose ratio of tacrolimus; AUC-MPA: mycophenolic acid area under the curve.

**Table 2:** Spearman correlation analysis between continuous variables and TTV load at month 1.

	rho	*p*
Recipient age (years)	−0.066	0.637
Time in renal replacement therapy (months)	0.052	0.708
Donor age (years)	0.123	0.376
HLA mismatches	−0.168	0.228
Cold ischemia time (hours)	0.088	0.526
First month eGFR (mL/min/1.73 m^2^)	−0.207	0.134
First month albuminuria (mg/g)	−0.158	0.283
Month 1 Prednisone dose (mg)	0.008	0.955
TTR 8–12 at month 1 (%)	−0.060	0.667
TTR > 12 at month 1 (%)	−0.012	0.931
Mean tacrolimus level at month 1 (ng/mL)	−0.070	0.617
CV at month 1 (%)	−0.016	0.909
C/D at month 1	−0.142	0.306
Mycophenolic acid trough level at month 1	0.223	0.116

eGFR: estimated glomerular filtration rate; TTR: time in therapeutic range; CV: coefficient of variability of tacrolimus; C/D: concentration/dose ratio of tacrolimus.

**Table 3:** Spearman correlation analysis between continuous variables and TTV load at month 3.

	rho	*p*
Recipient age (years)	−0.066	0.637
Time in renal replacement therapy (months)	0.052	0.708
Donor age (years)	0.328	0.015
HLA mismatches	−0.168	0.228
Cold ischemia time (hours)	−0.014	0.919
Third month eGFR (mL/min/1.73 m^2^)	−0.113	0.426
Third month albuminuria (mg/g)	0.000	0.999
Month 3 Prednisone dose (mg)	0.215	0.125
TTR 8–12 at month 3 (%)	−0.211	0.125
TTR > 12 at month 3 (%)	0.062	0.656
Mean tacrolimus level at month 3 (ng/mL)	−0.070	0.615
CV at month 3 (%)	0.105	0.451
C/D at month 3	−0.026	0.855
AUC-MPA at month 3	0.060	0.680

eGFR: estimated glomerular filtration rate; TTR: time in therapeutic range; CV: coefficient of variability of tacrolimus; C/D: concentration/dose ratio of tacrolimus; AUC-MPA: mycophenolic acid area under the curve.

## Data Availability

The data presented in this study are available on request from the corresponding author.
